# Preoperative CTC-Detection by CellSearch^®^ Is Associated with Early Distant Metastasis and Impaired Survival in Resected Pancreatic Cancer

**DOI:** 10.3390/cancers13030485

**Published:** 2021-01-27

**Authors:** Harald Hugenschmidt, Knut Jørgen Labori, Elin Borgen, Cathrine Brunborg, Cecilie Bendigtsen Schirmer, Lars Thomas Seeberg, Bjørn Naume, Gro Wiedswang

**Affiliations:** 1 Institute of Clinical Medicine, University of Oslo, 0424 Oslo, Norway; uxknab@ous-hf.no (K.J.L.); bna@ous-hf.no (B.N.); 2 Department of Transplantation Surgery, Oslo University Hospital, 0424 Oslo, Norway; 3 Department of Gastrointestinal Surgery, Oslo University Hospital, 0424 Oslo, Norway; sbseel@siv.no (L.T.S.); uxgrie@ous-hf.no (G.W.); 4 Department of Pathology, Oslo University Hospital, 0424 Oslo, Norway; ebg@ous-hf.no (E.B.); cbb@ous-hf.no (C.B.S.); 5 Oslo Centre for Biostatistics and Epidemiology, Oslo University Hospital, 0424 Oslo, Norway; uxbruc@ous-hf.no; 6 Department of Gastrointestinal Surgery, Vestfold Hospital Trust, 3103 Tønsberg, Norway; 7 Department of Oncology, Oslo University Hospital, 0424 Oslo, Norway

**Keywords:** pancreatic cancer, recurrence, metastasis, surgical resection, circulating tumour cells, CTC, CellSearch^®^, prognostication

## Abstract

**Simple Summary:**

The survival after surgical removal of pancreatic cancer is remaining dismal with frequent cases of treatment failure by early cancer recurrence. There are currently no means to preoperatively identify those at risk for early recurrence. Circulating tumour cells (CTCs) have been shown to identify high-risk individuals in a variety of cancer types. We did explore the impact of CTC detection with the FDA-approved CellSearch^®^ test on relapse and survival after removal of pancreatic cancer by surgery. CTCs were detected in about 7% of patients. The presence of CTCs in samples taken before the operation was associated with earlier cancer metastasis and shorter survival. The survival impact of CTCs was comparable to or exceeded the risk-factors that become available only after the operation like spread to lymph nodes or aggressive tissue growth patterns. We conclude that the detection of CTCs by this method warrants further exploration of its clinical application in presumed operable pancreatic cancer.

**Abstract:**

In patients with presumed pancreatic ductal adenocarcinoma (PDAC), biomarkers that may open for personalised, risk-adapted treatment are lacking. The study analysed the impact of CTCs-presence on the patterns of recurrence and survival in 98 patients resected for PDAC with 5–10 years of follow-up. Preoperative samples were analysed by the CellSearch^®^ system for EpCAM+/DAPI+/CK+/CD45-CTCs. CTCs were detected in 7 of the 98 patients. CTCs predicted a significantly shorter median disease-free survival (DFS) of 3.3 vs. 9.2 months and a median cancer specific survival (CSS)of 6.3 vs. 18.5 months. Relapse status was confirmed by imaging for 87 patients. Of these, 58 patients developed distant metastases (DM) and 29 developed isolated local recurrence (ILR) as the first sign of cancer relapse. All patients with CTCs experienced DM. pN-status and histological grade >2 were other independent risk factors for DM, but only CTCs predicted significantly shorter cancer-specific, disease-free and post-recurrence survival. Preoperative parameters did not affect clinical outcome. We conclude that CTC presence in resected PDAC patients predicted early distant metastasis and impaired survival. Preoperative CTCs alone or in combination with histopathological factors may guide initial treatment decisions in patients with resectable PDAC in the future.

## 1. Introduction

The prognosis of pancreatic ductal adenocarcinoma (PDAC) is dismal. The mortality to incidence ratio is 0.96, one of the highest among solid tumours [1,2]. Patient outcomes have remained virtually unchanged in the last decades [3,4], despite improvements in chemotherapy, radiation and operative technique. Surgical resection in cases of localised tumours is the only treatment offering potential cure, although the 5-year survival rate is between 5–9% in unselected cohorts [3]. About half of the patients experience recurrence within the first year [5,6]. There is no curative treatment option in the case of PDAC recurrence, although aggressive treatment may still prolong survival, especially in cases of isolated locoregional recurrence [7,8]. Currently, risk assessment from preoperatively available factors has shown limited ability to predict survival [9], but postoperative staging based on histopathological parameters can better predict impaired survival [7,10,11]. While prognostic factors available after surgery may be of value for better care, the current lack of individualised up-front treatment stratification by preoperative risk assessment is a major hindrance for improved treatment results of patients with presumed resectable PDAC. The preoperative presence of even one single circulating tumour cell (CTC) per sample in peripheral blood detected by the CellSearch^®^ system has been shown to be associated with metastatic recurrence and impaired survival in several cancer types [12,13]. Previous studies of PDAC-patients have shown CTCs to be a risk factor for impaired survival [14,15,16,17]. CTCs in the portal venous blood have been associated with liver metastasis in PDAC [18,19,20], but evidence linking CTCs from peripheral blood to a specific recurrence type is missing. The scope of the present study was to identify risk-factors for the type of recurrence and survival among patients that underwent a curative resection for PDAC with special focus on CTC-status.

## 2. Results

### 2.1. Patient Group Characteristics

Of the 98 patients in the study group, 95 patients had pT3 tumours, 73 were lymph node positive, 61 had R1 resections performed and 97 were of the pancreatobiliary histological subtype. Further details on clinical features, surgical procedures and systemic treatment of the study group are summarised in Table 1.

The 90-day mortality was 2.0% (2/98), both cases due to early cancer recurrence. The median observation time was 96 months (range 63–126).

**Figure 1:**
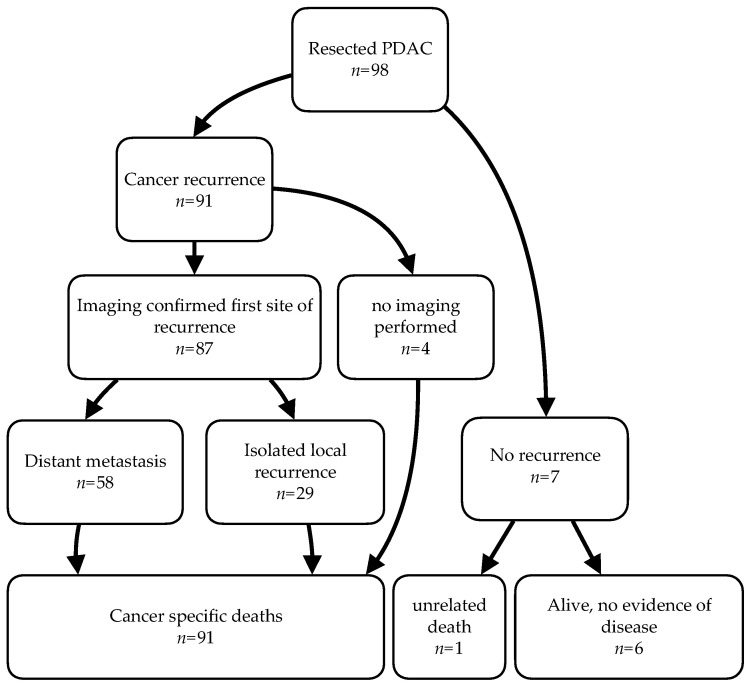
Overview of clinical events for the patient cohort, starting at the time of operation until the outcome stated in the last row at the end-point of observation was reached. **PDAC**: pancreatic ductal adenocarcinoma.

Figure 1 summarises the recurrence events including death during the follow-up period. Six (6.1%) patients were alive at the end of the study. Four patients died of cancer progression without the confirmation of the site of first recurrence, one patient died of pneumonia without signs of cancer recurrence. Among the 87 cases with imaging-confirmed recurrence, distant metastasis (DM) was detected first in 58 (67%) cases, 16 of whom had simultaneous local recurrence. Twenty-nine patients (33%) presented with isolated local recurrence (ILR). Detailed information on the distribution of clinicopathological parameters according to the recurrence status can be found in Table A1.

### 2.2. Distribution of CTCs and Site of First Recurrence

**Figure 2:**
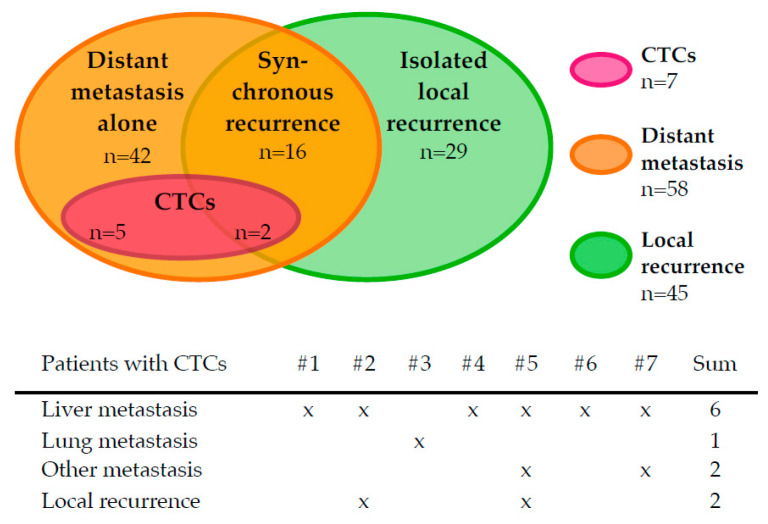
Distribution of circulating tumour cell (CTC)-positive cases in the study group by location of first recurrence.

The frequency of CTCs in the entire group was 7.1% (7/98) (Table 1). In six of these patients, one CTC per sample was detected, one patient had 33 CTCs (see Table A2). As shown in Figure 2, all CTC-positive patients developed distant metastasis first. Furthermore, six of seven CTC-positive patients developed liver metastasis. Two patients with CTCs had both distant metastasis and local recurrence concurrently. No CTCs were observed in patients with ILR.

#### 2.2.1. Recurrence and Survival Analysis

**Figure 3:**
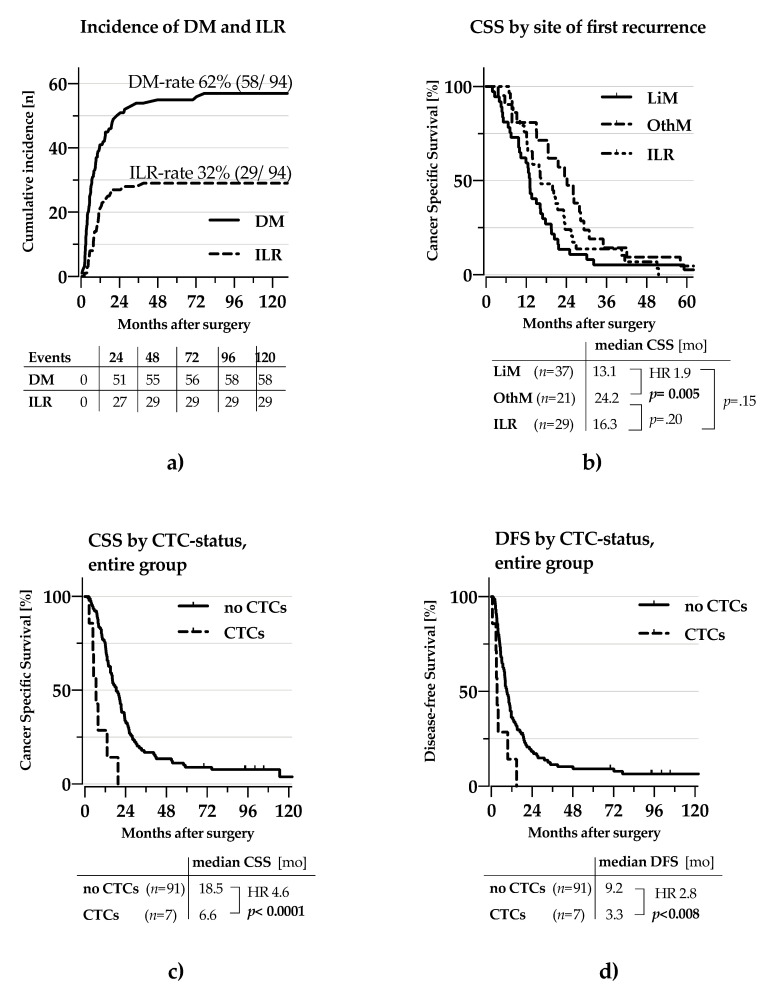
Recurrence and Survival patterns for resected PDAC Kaplan-Meier curves for survival and time to recurrence. (**a**) Incidence by site of first recurrence (**b**) Cancer specific survival by site of first recurrence. (**c**) CSS by CTC-status for all patients (**d**) DFS by CTC-status for all patients, CSS: cancer-specific survival, DFS: disease-free survival, DM: distant metastasis ILR: islolated local recurrence, LiM: Liver metastasis, alone or in combination with others, OthM: any other type of distant metastasis. HR: Hazard ratio. Statistical significance assumed for *p* < 0.05, indicated by use of bold font.

While the incidence of DM was twice the incidence of ILR (58 vs 29 patients), the time to either type of recurrence was not significantly different (TDM 6.2 months vs. TILR 9.2 months *p* = 0.886; Figure 3a). Thirty-seven patients experienced metastases to the liver, which was the most frequent localisation of recurrence. Liver metastasis was associated with a significantly shorter CSS compared to distant metastases at other sites (13.1 vs. 24.2 months, HR 1.9 *p* = 0.005; Figure 3b). The differences in survival between any of these two groups and patients with ILR did not reach statistical significance (Figure 3b). Finally, CTCs had strong impact on CSS (6.6 vs. 18.5 months, HR 4.6 *p* < 0.001; Figure 3c) and DFS (3.3 vs. 9.2 months, HR 2.8 *p* = 0.008; Figure 3d).

Uni- and multivariable analysis of risk-factors for either TDM, TILR or CSS identified several independent risk factors (Table 2).

CTC-status, pN and histological grade > 2 were independent risk factors for TDM. R-status was the sole risk factor to predict TILR. In addition, CTC-status and pN were independent risk factors for CSS (Table 2).

**Figure 4:**
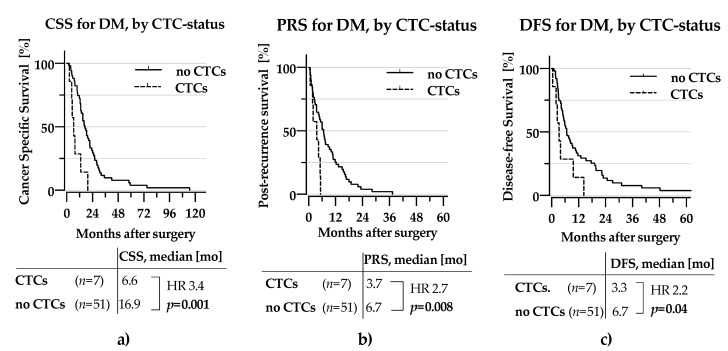
KM-curves for patients with DM as site of first metastasis (**a**) CSS by CTC-status (**b**) PRS by CTC-status (**c**) DFS by CTC-status **CTC**: Circulating tumour cell, **CSS**: Cancer-specific survival, PRS: Post-recurrence survival, DFS: Disease-free survival, HR: Hazard ratio. Statistical significance assumed for *p* < 0.05, indicated by use of bold font.

Figure 4 presents the isolated survival results for patients who developed distant metastasis during the observation period, showing reduced CSS and DFS for CTC-positive patients (Figure 4a,c). Also, the post-recurrence survival was severely affected by CTC-status (HR 2.7, *p* = 0.008, Figure 4b).

#### 2.2.2. CSS According to Subgroups by Combining Risk-factors for Distant Metastasis

**Figure 5:**
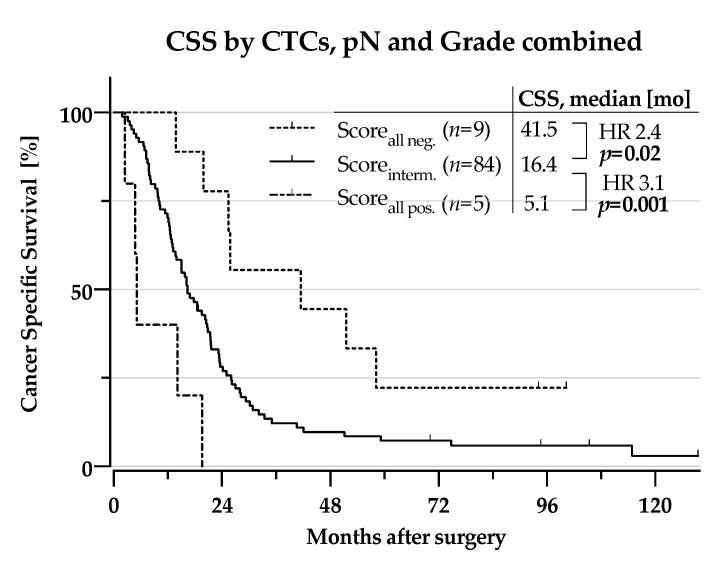
Kaplan-Meier curves for survival times: Cancer specific survival by combination of CTCs, pN1 and Grade > 2 into a score for the entire cohort. Score_all neg__._: all three negative, Score_interm._: one or two factors positive, Score_all pos__._: all three positive, CSS: cancer-specific survival, CTC: circulating tumour cell, HR: Hazard ratio. Statistical significance assumed for *p* < 0.05, indicated by use of bold font.

When combining the three independent unfavourable risk factors for metastatic recurrence (CTC-positivity, node-positivity and high histologial grade), patients having all three factors had a dismal CSS compared to those positive for just one or two of the factors (5.1 months vs. 16.4 months, HR 3.1, *p* = 0.001, Figure 5).

Interestingly, those with none of the risk factors present had a markedly improved CSS compared to those with one or two factors present (41.5 months vs. 16.4 months, HR 2.4, *p* = 0.02, Figure 5).

## 3. Discussion

This report presents results from a well-characterised single institution study of patients resected for PDAC with an extended observation time. To the best of our knowledge, the cohort represents currently the largest group of resected PDAC patients analysed for CTCs by CellSearch^®^. The study gives insight into the association between preoperative CTC-status, other clinical and histopathological risk factors and the patterns of recurrence and patient survival.

The detection threshold of ≥1 CTC/7.5 mL was chosen for the present study in accordance with the previous publication from the extended cohort [17] and other CellSearch^®^-based studies in pancreatic cancer [14,21,22,23,24,25]. While the lower threshold of ≥1 CTC/7.5 mL is close to the limits of the detection technology [26,27], there is further evidence from well controlled studies of non-metastatic patients with cancer diagnoses [28,29,30] supporting the use the 1 CTC/7.5 mL threshold, too. For advanced cancer patients, a higher threshold of ≥2–3 CTC/7.5 mL is well established [31].

The frequency of CTC-detection varies between detection technologies, both due to differences in enrichment and detection efficiency [23,32,33] but also due to biological differences between subtypes of CTCs [34,35,36]. Up to 60% CTC-positivity with survival impact for resectable PDAC have been reported, but the methods seem to be not ready for clinical application in the near future [34,35]. Improvements in the methodologies are expected to come. Currently, however CellSearch^®^ was ranked above other methods of immunodetection and PCR in a systematic comparison of methods [37].

CTCs in the current study predicted earlier metastasis and impaired survival following potentially curative surgery. The relatively low frequency of CTCs of 7.1% for the 98 patients in the study group is consistent with other reports [18,21,24,38] utilising the same detection method. The CirCe07-trial reported 5% and 9% CTCs in Stage III PDAC before and after neoadjuvant treatment, respectively. These results in the same range as our results of 7.1% in our cohort of mainly stage IIb-patients. An increased CTC-count was reported in the portal venous blood from two smaller cohorts of resectable PDAC [18,39]. While CTC-presence in the portal venous blood was associated with liver metastasis in these studies, we are not aware of any reports of an association between CTCs from peripheral blood and recurrence types. The presented association of CTCs with DM as the first site of recurrence and the prediction of a markedly shorter TDM underscore the usefulness of CTCs for prediction of early metastatic events in the clinic. In addition, of all risk-factors examined, only CTCs had a significant impact also on post-recurrence survival, which further corroborates the severe impact of CTC-presence for the subsequent CSS. Of the other risk factors with impact on DM, pN-status and high histological grade, only pN-status correlated with CSS and none with PRS. Still, a combination of pN-status, grade and CTCs allowed marked differentiation of survival among the patients.

The ability to preoperatively predict the likelihood for long-term survival from factors identified in the initial work-up would be of potential clinical benefit for patients currently scheduled for up-front surgical resection. Recently, a nomogram based solely on preoperative variables independently associated with overall survival among 7849 patients (CA 19–9, neoadjuvant therapy, tumour size, age, centre volume, Charlson-Deyo score, primary site, sex) has been reported [9]. Although the HRs of the single risk factors were very low (ranging from 1.07–1.37) the cumulative score from these parameters was able to classify patients into three groups of favourable (>2.5 y median OS), intermediate (1.5–2.5 y median OS) and poor prognosis (<1.5 y median OS). In contrast, in the present study the HR for CTCs as single parameter was 3.7 (95% CI 1.7–8.3, *p* = 0.001), with a median CSS of 6.63 months for CTC-positive patients. This supports CTCs to provide added clinical value to the information from the nomogram. However, “in-between study comparisons” should be interpreted with caution. A validated postoperative survival prediction model based on tumour grade, lymph node ratio, margin status and adjuvant therapy (the Amsterdam prediction model) has also been reported [40,41]. While of interest for the subsequent handling of the individual patient after surgery, the clinical utility of this score may be limited by the lack of impact on preoperative decisions. Concerning adjuvant treatment, there has been progress in recent years driven by advances in chemotherapy regimens [42]. Further personalisation by CTC-based risk assessment would be an avenue to future improvement. The prognostic advantage of patients with no CTC; pN0 and grade ≤2 warrants further exploration as to whether these patients would benefit from adjuvant therapy.

The current study confirms the prognostic impact of histopathological factors as lymph node status, margin status and histological grade, but adds important information on the potential use of preoperative CTC-analysis for prognostic classification of PDAC. With the currently available techniques for CTC-detection and treatment modalities in PDAC the most promising use of CTC-analysis probably would be to support surgical treatment decisions.

Studies exploring the prognostic value of ctDNA in PDAC have the potential to substantially enhance the information from a preoperative blood sample [43,44,45,46]. There is evidence from comparative studies that ctDNA can be detected in higher frequency than CTCs, but the prognostic impact of different mutational signatures in ctDNA is not fully resolved [21,47,48]. As reported in a recent meta-analysis, ctDNA was detected preoperatively in 8.3–68.6% of resectable PDAC patients and predicted CSS with a HR of 2.27 (95% CI 1.13–4.56) [43]. Most probably, future improvements in systemic treatment are dependent upon identification of the core molecular characteristics or driver mutations of the cancer cells.

Limitations of the present study are mainly due to the low detection frequency for CellSearch^®^ in the setting of presumed resectable PDAC. Even as the study presents the largest cohort of PDAC examined by this method, the low detection frequency of around 7% and a still low number of patients studied limits the generalisation of the results reported. An independent study confirmation of the prognostic value of CTCs by CellSearch^®^ would strongly support the use of CTCs as a biomarker in future clinical practice. Ideally, further studies of the liquid-biopsy paradigm would also include detection methods with potentially complementary properties like ctDNA and exosomes to advance treatment outcome for this patient group.

## 4. Materials and Methods

### 4.1. Patients, Study Design and Follow-up

The cohort in the present study was recruited among patients referred to the hepatobiliary unit at Oslo University Hospital with the clinical suspicion of a potentially resectable solid mass in the periampullary region. Patients who were evaluated in a multidisciplinary tumour board as potentially resectable [49] were offered participation in an observational study on the survival impact of tumour cells in the peripheral blood (CTC) or bone marrow (DTC). Properties of the study cohort and general results have been published previously [17,50].

Prospective recording of the clinical data was done in an Epi-Info 3.5.3 database (CDC, Atlanta, GA, USA). Follow-up of patients included a thoracoabdominal CT-scan and CA19–9 assessment twice a year on a voluntary basis, either at the study hospital or the local hospital as previously described [51].

The type of recurrence was defined by the first location of cancer relapse as detected by diagnostic imaging. When imaging findings were consistent with recurrence, bioptic verification was rarely performed. Isolated local recurrence (ILR) was defined as recurrence solely in the remnant pancreas or in the surgical bed, such as soft tissue along the celiac or superior mesenteric artery, aorta or around the pancreaticojejunostomy site. Distant metastasis (DM) was defined as recurrence in any site other than ILR. In the case of concurrent metastatic and local recurrence, cases were assigned to the DM group due to the greater survival impact of metastases [52]. Starting point for the time-to-event variables TDM and TILR was the date of operation, until DM or ILR respectively. Mortality data were taken from the Norwegian Cause of Death Registry, provided by the Norwegian Institute of Public Health. Cancer Specific Survival (CSS) was defined the time from date of surgery until cancer related death, post-recurrence survival (PRS) as the time between recurrence until cancer related death and disease-free survival (DFS) as the time from surgery until any kind of recurrence or death. Cases reaching the end of observation without event were censored for these variables [53]. Patient inclusion started from1 October 2009, patient inclusion stopped 31 December 2014 and observation ended July 31st, 2020. Analysis and reporting were done observing the STROBE (2014) and REMARK (2012) checklists.

### 4.2. Characteristics of the Patient Cohort

For the present study, 98 patients in whom a curative resection for PDAC was successfully performed and the CTC-status was determined were selected from a cohort of 277 patients with presumed resectable periampullary carcinoma (results previously published [17]).

Along with the CTC-status, the following clinical risk factors were analysed: age, gender, preoperative CA19-9 level, tumour size on CT-scan, AJCC/UICC-stage (7th ed.), pTNM-staging including resection margin, cancer origin, tumour grade, histological subtype (predominantly intestinal or pancreatobiliary), vascular and perineural infiltration. CA19-09 and tumour size were dichotomized at the following thresholds: CA19-9 ≥ 200 kU/L and the size of the tumour on CT-scan ≥ 25 mm (for results, see Table 1).

### 4.3. CTC Detection

The detection of EpCAM-positive CTCs was performed on the FDA-approved CellSearch^®^ system, utilising the Circulating Epithelial Cell Kit (Product Code: 7900000), the CellTracks^®^ Autoprep^®^ system (Product Code: 9541) and the CellTracks Analyzer ll^®^ system (Product Code: 9555, all items by Menarini Silicon Biosystems Inc., Castel Maggiore, Italy) as follows:

Blood samples of 7.5 mL were drawn into CellSave^®^ tubes (Product Code: 7900005) just before surgery (one tube per patient). The samples were kept at room temperature until processing within 72 h at the Micrometastasis Laboratory, Oslo University Hospital, Oslo, Norway. According to the manufacturer’s specifications, blood (7.5 mL) from the CellSave tube was transferred to a 15 mL tube, added 6.5 mL CellSearch^®^ dilution buffer (included within the Circulating Epithelial Cell Kit) and centrifuged for 10 min at 800× *g*. The tube was then placed in the automated Celltracks Autoprep instrument where CTCs are immuno-magnetically separated from the other cells in the blood by ferrofluid nanoparticles coated with anti-EpCAM antibodies, followed by immunostaining of CTC with anti-cytokeratin (anti-CK8, 18 and 19; PE-marked) and anti-CD45 (APC marked) together with DAPI nuclear staining. Within the system the CTC-enriched sample is transferred to a cartridge localized inside a magnetic holder (“Magnest”). Within the magnetic field the captured cells migrate to the analytical plane of the cartridge. The holder with the cartridge is subsequently transferred to the Celltracks Analyzer ll^®^ System where the cells are scanned in 4 channel fluorescence microscopy and candidate detected elements are presented on a screen for review and classification by the user.

Detected elements were reviewed by a certified technician (CS) and diagnoses of CTCs confirmed by an experienced pathologist (EB) according to standardized criteria. A threshold of 1 CTC/7.5 mL was set for a patient sample to be considered CTC-positive in accordance with previously published reports [17]. Results were stored in the National Micrometastasis Register at the Micrometastasis Laboratory, Department of Pathology, Oslo University Hospital, and were not available to treating clinicians. Following closure of the observation period, CTC-results were extracted from the Micrometastasis register and combined with the clinical data.

### 4.4. Statistics

Analyses were carried out in SPSS, V26 (IBM Cooperation Analytics, Armonk, NY, USA) and STATA 16 (Stata Corp LLC, 4905 Lakeway Drive College Station, TX, USA). Graphs were prepared in PRISM 8 (GraphPad Software Inc., La Jolla, CA, USA). Survival analyses were computed by the Kaplan-Meier method, the difference of curve pairs was assessed by the Log-rank test. The adjustment for confounders was performed by Cox Proportional Hazard regression models with a manual backward stepwise elimination procedure. Multivariable analyses were preceded by estimation of correlation between confounders. The Proportional Hazard assumptions was controlled by plotting the logarithm of the integrated hazards (log–log survival plots). The association between potential risk factors and survival metrics was quantified by hazard ratios (HR) with a 95% confidence interval (95% CI). For *p* < 0.05, statistical significance was assumed.

## 5. Conclusions

CTCs in resected PDAC predict early distant metastasis and markedly impaired survival. Preoperative CTC detection, alone or in combination with histopathological factors, may be used to guide risk-adapted treatment in patients with presumed resectable PDAC.

## Figures and Tables

**Table 1:** Clinicopathological parameters for resected PDAC (*n* = 98).

Demographics	*n*	(%)
Age, median [years]	68	(34–80)
Sex, male	50	(51.0%)
**Preoperative Risk Factors**		
CTCs ≥ 1/7.5 mL	7	(7.1%)
CA19–9 ≥ 200 kU/l	31/74	(41.9%) *24 missing*
Tumour size on imaging> 25 mm	39/97	(40.2%) *1 missing*
Bilirubin > 50 µmol/L	75/94	(79.8%) *4 missing*
**Treatment**		
Neoadjuvant chemotherapy:		
GEM	3	(3.0%)
FOLFIRINOX	1	(1.0%)
Operation:		
PPPD	77	(78.6%)
PD	13	(13.3%)
Total pancreatectomy	8	(8.2%)
Venous resection	36	(36.7%)
Adjuvant chemotherapy:		
FLV	53	(54.1%)
GEM	6	(6.1%)
FLOX	3	(3.1%)
none	36	(36.7%)
**Histopathologic results**		
Pancreatobiliary type	97	(99%)
Intestinal type	1	(1.0%)
UICC-stage (V7):		
Ib	2	(2.0%)
IIa	23	(23.5%)
IIb	73	(74.5%)
pN1-status	73	(74,5%)
R1-status	61	(62.2%)
Vascular infiltration	65	(66.3%)
Perineural infiltration	91	(92.9%)

FLV: 5-fluorouracil and folinic acid; FLOX: 5-fluorouracil, folinic acid and oxaliplatine; FOLFIRINOX: 5-fluorouracil, folinic acid, irinotecan and oxaliplatin; GEM: gemcitabine; PD: pancreatoduodenectomy; PPPD: pylorus preserving PD.

**Table 2:** Univariate and multivariable analyses of time to type of first recurrence and cancer specific survival.

Potential Risk Factors			TDM				TILR					CSS		
	Level	*n*	Univariate HR (95% CI)	*p*	Multivariable HR (95% CI)	*p*	Univariate HR (95% CI)	*p*	Multivariable HR (95% CI)	*p*	Univariate HR (95% CI)	*p*	Multivariable HR (95% CI)	*p*
**Age**	≤70 >70	58 40	1.2 (0.7–2.1)	0.602			1.3 (0.6–2.8)	0.449			1.4 (0.9–2.1)	0.156		
**Sex**	Male female	50 48	0.7 (0.4–1.2)	0.127			0.8 (0.4–1.7)	0.627			0.8 (0.5–1.2)	196		
**CA19–9** **(*24 missing*)**	≥200 <200	43 31	1.2 (0.6–2.2)	0.623			1.0 (0.5–2.3)	0.939			1.1 (0.7–1.8)	0.601		
**Tumour sizeon CT** **(*1 missing)***	≥25 mm <25 mm	39 58	1.4 (0.8–2.3)	0.272			2.0 (1.0–4.)	0.057			1.3 (0.8–2.0)	0.241		
**CTC**	≥1 none	7 91	**3.9** **(1.7–8.8)**	**0.001**	**2.9** **(1.3–6.6)**	**0.010**	All censored				**4.4** **(2.0–9.8)**	**<0.001**		**0.001**
**Neoadjuvant chemotherapy**	Yes no	4 94	1.2 (0.3–5.0)	0.928			2.0 0.5–8.7)	0.333			1.0 (0.4–3.7)	0.964		
**Venous resection**	yes no	36 62	**0.5** **(0.3–0.9)**	**0.017**	eliminated		1.6 (0.8–3.3)	0.209	eliminated		0.8 (0.5–1.2)	0.326	eliminated	
**Adjuvant chemotherapy**	Yes no	62 36	0.8 (0.5–1.4)	0.466			0.9 (0.4–1.9)	0.735			0.7 (0.7–1.1)	0.124		
**pT**	1,2 3,4	3 95	0.7 (0.2–2.7)	0.557			All censored				0.5 (0.1–1.9)	0.300		
**pN**	1 0	73 25	**3.6** **(1.7–7.3)**	**0.001**	**3.0** **(1.5–6.3)**	**0.003**	1.6 (0.7–3.6)	0.226	eliminated		**2.1** **(1.2–3.5)**	**0.004**	**2.0** **(1.2–3.4)**	**0.009**
**Grade**	G3,4 G1,2	28 70	**2.1** **(1.2–3.6)**	**0.007**	**1.8** **(1.1–3.1)**	**0.030**	0.6 (0.2–1.7)	0.336	eliminated		1.4 (0.9–2.1)	0.184	eliminated	
**R**	1 0	61 37	1.4 (0.8–2.3)	0.212	eliminated		**2.3** **(1.0–5.0)**	**0.040**	**2.3** **(1.0–5.0)**	**0.040**	1.4 (0.9–2.2)	0.128	eliminated	
**Vascular infiltration**	1 0	65 33	1.7 (0.9–3.1)	0.075	eliminated		0.9 (0.4–1.9)	0.790	eliminated		**1.6** **(1.0–2.6)**	**0.034**	eliminated	
**Perineural infiltration**	1 0	91 7	1.3 (0.5–3.7)	0.588			2.7 (0.4–19.7)	0.334			1.8 (0.7–4.6)	0.185		

Uni- and multivariable analysis of prognostic factors for TDM/TILR of resected PDAC patients. Univariate HR: Cox regression using single factors; Multivariable HR: Cox regression model with manual backwards elimination. Statistical significance assumed for *p* < 0.05, indicated by use of bold font. HR: Hassard ratio. CI: Confidence interval. CSS: cancer-specific survival, TDM: time to distant metastasis. TILR: time to isolated local recurrence.

## Data Availability

The data presented in this study are available on reasonable request from the corresponding author. The data are not publicly available due to privacy regulations.

## Appendix A

**Table A1:** Clinical parameters by site of first relapse.

	Entire Cohort (*n* = 98)	Remission (*n* = 6)	ILR (*n* = 29)	DM (*n* = 58)	No Imaging (*n* = 4)
**Demographics**					
**Age, median [years]**	68 (34–80)	65 (57–75)	68 (51–78)	68 (34–79)	74 (68–80)
**Sex, male**	50 (51.0%)	2 (33.3%)	14 (48.3%)	33 (59.0%)	0 (0%)
**Preoperative Risk Factors**					
**CTCs ≥ 1**	7 (7.1%)	none	none	7 (12.5%)	none
**CA19-9 ≥ 200 kU/l**	31/74 (41.9%) *24 missing*	2/4 (50%) *2 missing*	10/25 (40%) *2 missing*	18/42 (42.9%) *14 missing*	½ (50%) *2 missing*
**Tumour size on imaging> 25 mm**	39/97 (40.2%) *1 missing*	2 (33.3%)	15 (51.7%)	21/57 (36.8%) *1 missing*	1 (25%)
**Bilirubin > 50 µmol/L**	75/94 (79.8%) *4 missing*	5 (83.3%)	20/27 (74.1%) *2 missing*	47/56 (82.4%) *1 missing*	2 (50%)
**Treatment**					
**Neoadjuvant therapy:**					
GEM	3(3.0%)	None	2(6.9%)	1(1.7%)	None
FOLFIRINOX	1 (1.0%)	none	none	1(1.7%)	none
**Operation:**					
PPPD	77 (78.6%)	5 (83.3%)	23 (79.3%)	46 (79.3%)	2 (50%)
PD	13 (13.2%)	1 (16.7%)	4 (13.8%)	7 (12.1%)	1 (25%)
Tot. pancreatectomy	8 (8.2%)	none	2 (6.9%)	5 (8.6%)	1 (25%)
Venous resection	36 (36.7%)	3 (50%)	16 (55.2%)	15 (25.9%)	1 (25%)
**Adjuvant therapy:**					
FLV	53 (54.1%)	5 (83.3%)	14 (48.3%)	31 (53.4%)	2 (50%)
GEM	6 (6.1%)	None	3 (10.3%)	3 (5.2%)	None
FLOX	3 (3.1%)	None	1 (3.4%)	1 (1.7%)	None
none	36 (36.7%)	1 (16.7%)	11 (37.9%)	22 (37.9%)	2 (50%)
**Histopathologic results**					
**Pancreatobiliary type**	97 (99%)	6	28 (96.6%)	58 (100%)	4 (100%)
**Intestinal type**	1 (1%)	-100%	1 (3.4%)		
**UICC-stage (V7):**					
Ib	2 (2.0%)	None	None	1 (1.7%)	0 (0%)
IIa	23 (23.5%)	4 (66.7%)	9 (31.0%)	8 (13.8%)	2 (50%)
IIb	73 (74.5%)	2 (33.3%)	20 (69.0%)	49 (84.5%)	2 (50%)
**pN1-status**	73 (74,5%)	2 (33.3%)	20 (69.0%)	49 (84.5%)	2 (50%)
**R1-status**	61 (62.2%)	3 (50%)	20 (69.0%)	35 (60.3%)	3 (75%)
**Vascular infiltration**	64 (65.3%)	2 (33.3%)	17 (55.1%)	42 (72.4%)	3 (75%)
**Perineural infiltration**	91 (92.9%)	4 (66.7%)	28 (96.6%)	54 (93.1%)	4 (100%)

Comparison of characteristics between different subgroups and the entire cohort. One single patient with death unrelated to cancer omitted from the subgroup presentation. ILR: isolated locoregional recurrence, DM: distant metastasis, CTC: circulating tumour cell, FLV: 5-fluorouracil and folinic acid; FLOX: 5-fluorouracil, folinic acid and oxaliplatine; FOLFIRINOX: 5-fluorouracil, folinic acid, irinotecan and oxaliplatin; GEM: gemcitabine; PD: pancreato-duodenectomy, PPPD: pylorus preserving PD.

**Table A2:** Clinicopathological parameters and survival of patients with CTCs.

Age	Sex	UICC-Stage	pT	pN	M	G	R	VI	PNI	CTCs/ 7.5 mL	CSS [mo]	DFS [mo]
**79**	m	St. IIb	3	1	0	4	0	1	1	33	2.5	0.5
**65**	m	St. IIb	3	1	0	3	0	1	1	1	14.1	9.7
**71**	m	St. IIb	3	1	0	2	1	0	1	1	7.8	2.5
**70**	m	St. IIb	3	1	0	2	1	1	1	1	19.6	14.3
**69**	m	St. IIb	3	1	0	2	1	0	1	1	6.6	2.0
**76**	f	St. IIb	3	1	0	2	1	1	1	1	4.7	3.9
**57**	m	St. IIb	3	1	0	2	0	1	1	1	5.1	3.3

Demographics and basic clincopathological parameters of CTC-positive patients with CTC-number and survival metrics. VI: vessel infiltration; PNI: neural infiltration; CSS: cancer specific survival; DFS: disease-free survival.

## References

[1] Hariharan D., Saied A., Kocher H.M. (2008). Analysis of mortality rates for pancreatic cancer across the world. HPB (Oxford).

[2] Søreide K., Aagnes B., Møller B., Westgaard A., Bray F. (2010). Epidemiology of pancreatic cancer in Norway: Trends in incidence, basis of diagnosis and survival 1965–2007. Scand. J. Gastroenterol..

[3] Siegel R.L., Miller K.D., Jemal A. (2018). Cancer statistics, 2018. CA Cancer J. Clin..

[4] Larsen I.K., Møller B., Robsahm T.E., Johannsessen T.B., Grimsrud T.K., Larønningen S., Jakobsen E., Ursin G., Larsen I.K. (2019). Cancer in Norway 2018.

[5] Van den Broeck A., Sergeant G., Ectors N., Van Steenbergen W., Aerts R., Topal B. (2009). Patterns of recurrence after curative resection of pancreatic ductal adenocarcinoma. Eur. J. Surg. Oncol..

[6] Sperti C., Pasquali C., Piccoli A., Pedrazzoli S. (1997). Recurrence after resection for ductal adenocarcinoma of the pancreas. World J. Surg..

[7] Suto H., Okano K., Oshima M., Ando Y., Takahashi S., Shibata T., Kamada H., Kobara H., Masaki T., Suzuki Y. (2019). The predictors and patterns of the early recurrence of pancreatic ductal adenocarcinoma after pancreatectomy: The influence of pre- and post- operative adjuvant therapy. BMC Surg..

[8] Groot V.P., van Santvoort H.C., Rombouts S.J.E., Hagendoorn J., Borel Rinkes I.H.M., van Vulpen M., Herman J.M., Wolfgang C.L., Besselink M.G., Molenaar I.Q. (2017). Systematic review on the treatment of isolated local recurrence of pancreatic cancer after surgery; re-resection, chemoradiotherapy and SBRT. HPB (Oxford).

[9] Oba A., Croce C., Hosokawa P., Meguid C., Torphy R.J., Al-Musawi M.H., Ahrendt S., Gleisner A., Schulick R.D., Del Chiaro M. (2020). Prognosis Based Definition of Resectability in Pancreatic Cancer: A Road Map to New Guidelines. Ann. Surg..

[10] Groot V.P., Rezaee N., Wu W., Cameron J.L., Fishman E.K., Hruban R.H., Weiss M.J., Zheng L., Wolfgang C.L., He J. (2018). Patterns, Timing, and Predictors of Recurrence Following Pancreatectomy for Pancreatic Ductal Adenocarcinoma. Ann. Surg..

[11] Honselmann K.C., Pergolini I., Castillo C.F.-D., Deshpande V., Ting D., Taylor M.S., Bolm L., Qadan M., Wellner U., Sandini M. (2019). Timing but Not Patterns of Recurrence Is Different Between Node-negative and Node-positive Resected Pancreatic Cancer. Ann. Surg..

[12] Riethdorf S., O’Flaherty L., Hille C., Pantel K. (2018). Clinical applications of the CellSearch platform in cancer patients. Adv. Drug Deliv. Rev..

[13] Bidard F.C., Mathiot C., Delaloge S., Brain E., Giachetti S., de Cremoux P., Marty M., Pierga J.Y. (2010). Single circulating tumor cell detection and overall survival in nonmetastatic breast cancer. Ann. Oncol..

[14] Effenberger K.E., Schroeder C., Hanssen A., Wolter S., Eulenburg C., Tachezy M., Gebauer F., Izbicki J.R., Pantel K., Bockhorn M. (2018). Improved Risk Stratification by Circulating Tumor Cell Counts in Pancreatic Cancer. Clin. Cancer Res..

[15] Poruk K.E., Valero V., Saunders T., Blackford A.L., Griffin J.F., Poling J., Hruban R.H., Anders R.A., Herman J., Zheng L. (2016). Circulating Tumor Cell Phenotype Predicts Recurrence and Survival in Pancreatic Adenocarcinoma. Ann. Surg..

[16] Sergeant G., van Eijsden R., Roskams T., Van Duppen V., Topal B. (2012). Pancreatic cancer circulating tumour cells express a cell motility gene signature that predicts survival after surgery. BMC Cancer.

[17] Hugenschmidt H., Labori K.J., Brunborg C., Verbeke C.S., Seeberg L.T., Schirmer C.B., Renolen A., Borgen E.F., Naume B., Wiedswang G. (2020). Circulating Tumor Cells are an Independent Predictor of Shorter Survival in Patients Undergoing Resection for Pancreatic and Periampullary Adenocarcinoma. Ann. Surg..

[18] Bissolati M., Sandri M.T., Burtulo G., Zorzino L., Balzano G., Braga M. (2015). Portal vein-circulating tumor cells predict liver metastases in patients with resectable pancreatic cancer. Tumour. Biol..

[19] Tien Y.W., Kuo H.-C., Ho B.-I., Chang M.-C., Chang Y.-T., Cheng M.-F., Chen H.-L., Liang T.-Y., Wang C.-F., Huang C.-Y. (2016). A High Circulating Tumor Cell Count in Portal Vein Predicts Liver Metastasis from Periampullary or Pancreatic Cancer: A High Portal Venous CTC Count Predicts Liver Metastases. Medicine (Baltimore).

[20] Tao L., Su L., Yuan C., Ma Z., Zhang L., Bo S., Niu Y., Lu S., Xiu D. (2019). Postoperative metastasis prediction based on portal vein circulating tumor cells detected by flow cytometry in periampullary or pancreatic cancer. Cancer Manag. Res..

[21] Earl J., Garcia-Nieto S., Martinez-Avila J.C., Montans J., Sanjuanbenito A., Rodríguez-Garrote M., Lisa E., Mendía E., Lobo E., Malats N. (2015). Circulating tumor cells (Ctc) and kras mutant circulating free Dna (cfdna) detection in peripheral blood as biomarkers in patients diagnosed with exocrine pancreatic cancer. BMC Cancer.

[22] Kurihara T., Itoi T., Sofuni A., Itokawa F., Tsuchiya T., Tsuji S., Ishii K., Ikeuchi N., Tsuchida A., Kasuya K. (2008). Detection of circulating tumor cells in patients with pancreatic cancer: A preliminary result. J. Hepatobiliary Pancreat Surg..

[23] Khoja L., Backen A., Sloane R., Menasce L., Ryder D., Krebs M., Board R., Clack G., Hughes A., Blackhall F. (2012). A pilot study to explore circulating tumour cells in pancreatic cancer as a novel biomarker. Br. J. Cancer.

[24] Okubo K., Uenosono Y., Arigami T., Mataki Y., Matsushita D., Yanagita S., Kurahara H., Sakoda M., Kijima Y., Maemura K. (2017). Clinical impact of circulating tumor cells and therapy response in pancreatic cancer. Eur. J. Surg. Oncol..

[25] Bidard F.-C., Michiels S., Riethdorf S., Mueller V., Esserman L.J., Lucci A., Naume B., Horiguchi J., Gisbert-Criado R., Sleijfer S. (2018). Circulating Tumor Cells in Breast Cancer Patients Treated by Neoadjuvant Chemotherapy: A Meta-analysis. J. Natl. Cancer Inst..

[26] Terstappen L.W., Rao C., Gross S., Weiss A.J. (2000). Peripheral blood tumor cell load reflects the clinical activity of the disease in patients with carcinoma of the breast. Int. J. Oncol..

[27] Allard W.J., Matera J., Miller M.C., Repollet M., Connelly M.C., Rao C., Tibbe A.G.J., Uhr J.W., Terstappen L.W.M.M. (2004). Tumor cells circulate in the peripheral blood of all major carcinomas but not in healthy subjects or patients with nonmalignant diseases. Clin. Cancer Res..

[28] Rack B., Schindlbeck C., Jückstock J., Andergassen U., Hepp P., Zwingers T., Friedl T.W.P., Lorenz R., Tesch H., Fasching P.A. (2014). Circulating Tumor Cells Predict Survival in Early Average-to-High Risk Breast Cancer Patients. J. Natl. Cancer Inst..

[29] Janni W.J., Rack B., Terstappen L.W.M.M., Pierga J.-Y., Taran F.-A., Fehm T., Hall C., De Groot M.R., Bidard F.-C., Friedl T.W.P. (2016). Pooled Analysis of the Prognostic Relevance of Circulating Tumor Cells in Primary Breast Cancer. Clin. Cancer Res..

[30] van Dalum G., Stam G.-J., Scholten L.F.A., Mastboom W.J.B., Vermes I., Tibbe A.G.J., De Groot M.R., Terstappen L.W.M.M. (2015). Importance of circulating tumor cells in newly diagnosed colorectal cancer. Int. J. Oncol..

[31] Cabel L., Proudhon C., Gortais H., Loirat D., Coussy F., Pierga J.-Y., Bidard F.-C. (2017). Circulating tumor cells: Clinical validity and utility. Int. J. Clin. Oncol..

[32] Stoecklein N.H., Fischer J.C., Niederacher D., Terstappen L.W.M.M. (2016). Challenges for CTC-based liquid biopsies: Low CTC frequency and diagnostic leukapheresis as a potential solution. Expert Rev. Mol. Diagn..

[33] Brychta N., Drosch M., Driemel C., Fischer J.C., Neves R.P., Esposito I., Knoefel W., Möhlendick B., Hille C., Stresemann A. (2017). Isolation of circulating tumor cells from pancreatic cancer by automated filtration. Oncotarget.

[34] Poruk K.E., Blackford A.L., Weiss M.J., Cameron J.L., He J., Goggins M., Rasheed Z.A., Wolfgang C.L., Wood L.D. (2017). Circulating Tumor Cells Expressing Markers of Tumor-Initiating Cells Predict Poor Survival and Cancer Recurrence in Patients with Pancreatic Ductal Adenocarcinoma. Clin. Cancer Res..

[35] Gemenetzis G., Groot V.P., Yu J., Ding D., Teinor J.A., Javed A.A., Wood L.D., Burkhart R.A., Cameron J.L., Makary M.A. (2018). Circulating Tumor Cells Dynamics in Pancreatic Adenocarcinoma Correlate with Disease Status: Results of the Prospective CLUSTER Study. Ann. Surg..

[36] de Albuquerque A., Kubisch I., Breier G., Stamminger G., Fersis N., Eichler A., Kaul S., Stölzel U. (2012). Multimarker gene analysis of circulating tumor cells in pancreatic cancer patients: A feasibility study. Oncology.

[37] Stephenson D., Nahm C., Chua T., Gill A., Mittal A., de Reuver P., Samra J. (2017). Circulating and disseminated tumor cells in pancreatic cancer and their role in patient prognosis: A systematic review and meta-analysis. Oncotarget.

[38] Bidard F.C., Huguet F., Louvet C., Mineur L., Bouché O., Chibaudel B., Artru P., Desseigne F., Bachet J.B., Mathiot C. (2013). Circulating tumor cells in locally advanced pancreatic adenocarcinoma: The ancillary CirCe 07 study to the LAP 07 trial. Ann. Oncol..

[39] Buscail E., Alix-Panabières C., Quincy P., Cauvin T., Chauvet A., Degrandi O., Caumont C., Verdon S., Lamrissi I., Moranvillier I. (2019). High Clinical Value of Liquid Biopsy to Detect Circulating Tumor Cells and Tumor Exosomes in Pancreatic Ductal Adenocarcinoma Patients Eligible for Up-Front Surgery. Cancers.

[40] van Roessel S., Strijker M., Steyerberg E.W., Groen J.V., Mieog J.S., Groot V.P., He J., De Pastena M., Marchegiani G., Bassi C. (2020). International validation and update of the Amsterdam model for prediction of survival after pancreatoduodenectomy for pancreatic cancer. Eur. J. Surg. Oncol..

[41] Tol J.A.M.G., Brosens L.A.A., van Dieren S., van Gulik T.M., Busch O.R.C., Besselink M.G.H., Gouma D.J. (2015). Impact of lymph node ratio on survival in patients with pancreatic and periampullary cancer. Br. J. Surg..

[42] Turpin A., Amrani E.M., Bachet J.-B., Pietrasz D., Schwarz L., Hammel P. (2020). Adjuvant Pancreatic Cancer Management: Towards New Perspectives in 2021. Cancers.

[43] Lee J.-S., Rhee T.-M., Pietrasz D., Bachet J.-B., Laurent-Puig P., Kong S.-Y., Takai E., Yachida S., Shibata T., Lee J.W. (2019). Circulating tumor DNA as a prognostic indicator in resectable pancreatic ductal adenocarcinoma: A systematic review and meta-analysis. Sci. Rep..

[44] Hadano N., Murakami Y., Uemura K., Hashimoto Y., Kondo N., Nakagawa N., Sueda T., Hiyama E. (2016). Prognostic value of circulating tumour DNA in patients undergoing curative resection for pancreatic cancer. Br. J. Cancer.

[45] Sausen M., Phallen J., Adleff V., Jones S., Leary R.J., Barrett M.T., Anagnostou V., Parpart-Li S., Murphy D., Kay Li Q. (2015). Clinical implications of genomic alterations in the tumour and circulation of pancreatic cancer patients. Nat. Commun..

[46] Bettegowda C., Sausen M., Leary R.J., Kinde I., Wang Y., Agrawal N., Bartlett B.R., Wang H., Luber B., Alani R.M. (2014). Detection of circulating tumor DNA in early- and late-stage human malignancies. Sci. Transl. Med..

[47] Zhu Y., Zhang H., Chen N., Hao J., Jin H., Ma X. (2020). Diagnostic value of various liquid biopsy methods for pancreatic cancer: A systematic review and meta-analysis. Medicine (Baltimore).

[48] Kulemann B., Pitman M.B., Liss A.S., Valsangkar N., Fernández-Del Castillo C., Lillemoe K.D., Hoeppner J., Mino-Kenudson M., Warshaw A.L., Thayer S.P. (2015). Circulating tumor cells found in patients with localized and advanced pancreatic cancer. Pancreas.

[49] Labori K.J., Katz M.H., Tzeng C.W., Bjørnbeth B.A., Cvancarova M., Edwin B., Kure E.H., Eide T.J., Dueland S., Buanes T. (2016). Impact of early disease progression and surgical complications on adjuvant chemotherapy completion rates and survival in patients undergoing the surgery first approach for resectable pancreatic ductal adenocarcinoma—A population-based cohort study. Acta Oncol..

[50] Hugenschmidt H., Labori K.J., Brunborg C., Verbeke C.S., Seeberg L.T., Bendigtsen Schirmer C., Renolen A., Borgen E., Naume B., Wiedswang G. (2020). Cytokeratin-positive cells in the bone marrow from patients with pancreatic, periampullary malignancy and benign pancreatic disease show no prognostic information. BMC Cancer.

[51] Nordby T., Hugenschmidt H., Fagerland M.W., Ikdahl T., Buanes T., Labori K.J. (2013). Follow-up after curative surgery for pancreatic ductal adenocarcinoma: Asymptomatic recurrence is associated with improved survival. Eur. J. Surg. Oncol..

[52] Tao L., Zhang L., Peng Y., Tao M., Li L., Xiu D., Yuan C., Ma Z., Jiang B. (2016). Neutrophils assist the metastasis of circulating tumor cells in pancreatic ductal adenocarcinoma: A new hypothesis and a new predictor for distant metastasis. Medicine (Baltimore).

[53] Bonnetain F., Bonsing B., Conroy T., Dousseau A., Glimelius B., Haustermans K., Lacaine F., Van Laethem J.-L., Aparicio T., Aust D. (2014). Guidelines for time-to-event end-point definitions in trials for pancreatic cancer. Results of the DATECAN initiative (Definition for the Assessment of Time-to-event End-points in CANcer trials). Eur. J. Cancer.

